# Determination of Antimicrobial Resistance Megaplasmid-Like pESI Structures Contributing to the Spread of *Salmonella* Schwarzengrund in Japan

**DOI:** 10.3390/antibiotics14030288

**Published:** 2025-03-10

**Authors:** Kanako Ishihara, Suzuka Someno, Kaoru Matsui, Chisato Nakazawa, Takahiro Abe, Hayato Harima, Tsutomu Omatsu, Manao Ozawa, Eriko Iwabuchi, Tetsuo Asai

**Affiliations:** 1 Laboratory of Veterinary Public Health, Faculty of Agriculture, Tokyo University of Agriculture and Technology, 3-5-8 Saiwai-cho, Fuchu 183-8509, Tokyo, Japanmatsuika1341@gmail.com (K.M.); chisato.nakazawa.0218@gmail.com (C.N.); taka.vet.2024@gmail.com (T.A.);; 2 Center for Infectious Disease Epidemiology and Prevention Research, Tokyo University of Agriculture and Technology, 3-5-8 Saiwai-cho, Fuchu 183-8509, Tokyo, Japan; tomatsu@cc.tuat.ac.jp; 3 Assay Division I, National Veterinary Assay Laboratory, Ministry of Agriculture, Forestry, and Fisheries, 1-15-1 Tokura, Kokubunji 185-8511, Tokyo, Japan; manao_ozawa500@maff.go.jp; 4 Department of Nutrition, School of Nursing and Nutrition, Tenshi College, Kita 13 Higashi 3, Higashi-ku, Sapporo 065-0013, Hokkaido, Japan; iwabuti@tenshi.ac.jp; 5 United Graduate School of Veterinary Science, Gifu University, 1-1 Yanagido, Gifu-shi 501-1193, Gifu, Japan; asai.tetsuo.x3@f.gifu-u.ac.jp

**Keywords:** *Salmonella* Schwarzengrund, antimicrobial resistance, pESI, cgMLST

## Abstract

Background/Objectives: The acquisition of antimicrobial resistance by foodborne pathogens is a serious human health concern. In Japan, combinations of antimicrobial resistance genes in *Salmonella* from chicken meat were common among several serovars. Therefore, we hypothesized that different *S. enterica* serovars share a common antimicrobial resistance plasmid. Methods: Antimicrobial resistance transfer was tested in *S*. Infantis and *S*. Schwarzengrund, the major serovars used as donors. The plasmid structure was determined by subjecting *S*. Infantis Sal_238 and *S*. Schwarzengrund Sal_249 to short- and long-read sequencing. Results: The high homology between pSal_249Sch and pSal_238Inf suggests they have a common ancestor. Because the sequences of pSal_238Inf and pSal_249Sch were highly homologous to pESI (a plasmid for emerging *S.* Infantis), pSal_238Inf and pSal_249Sch were identified as pESI-like plasmids. *S*. Schwarzengrund is the third *Salmonella* serovar to expand its distribution related to pESI-like plasmid acquisition. Core-genome multilocus sequence-type analysis revealed that *S*. Schwarzengrund isolates with pESI-like plasmids from Japan (core-genome sequence-type [cgST] 167363 and cgST287831), the UK (cgST167363), and the USA (cgST167363, cgST196045, and cgST287831) were closely related; they are also suggested to share a common ancestor. The transfer of antimicrobial resistance was observed in combinations of both serovars. Specifically, the tentative plasmid sequence obtained via short-read sequencing, PCR, and conjugation experiments identified deletions of antimicrobial resistance genes (*aadA*, *sul1*, and *tetA*), class 1 integron, mercury resistance operon, and/or plasmid transfer region in the pESI-like plasmid. Conclusion: These data on the structural diversity of pESI-like plasmids suggest that some time has passed since *S*. Schwarzengrund acquired them.

## 1. Introduction

*Salmonella* spp. is a common cause of diarrheal diseases globally. Most diarrhea cases occur due to the consumption of contaminated food of animal origin [1]. Over 2500 *Salmonella* serovars have been identified to date [2]. Most serovars are found in various hosts. However, almost all serovars cause diseases in humans. *Salmonella* typically causes gastroenteritis, which often does not cause complications. Antimicrobial therapy is needed for some high-risk groups, such as infants, the elderly, and immunocompromised patients, but not for mild or moderate cases in healthy individuals [3].

In recent years, the prevalence of *Salmonella enterica* serovar Infantis (*S.* Infantis) infections has increased worldwide, owing to contamination of chicken and other foods. With multilocus sequence typing, *S*. Infantis sequence type (ST) 32 has significantly increased broiler production, leading to its spread through the poultry food chain [4]. *S*. Infantis isolates obtained from patients and chickens were resistant to multiple antimicrobials [4]. Plasmids are important vectors for the dissemination of antimicrobial resistance genes in bacteria. A conjugative megaplasmid named pESI (plasmid for emerging *S.* Infantis) was previously identified and characterized [5]. pESI had a significant impact on the spread of *S*. Infantis [5].

The area of *S*. Schwarzengrund-contaminated broiler chickens expanded from Western Japan to Eastern Japan in 2015 [6]. Since *S*. Schwarzengrund isolates obtained from chicken meat in Japan between 2008 and 2019 were assigned as ST241, *S*. Schwarzengrund ST241 may have spread from Western to Eastern Japan [7]. In a previous study, 157 *Salmonella* isolates were obtained from chicken meat samples collected between 2015–2017 [6]. Of these, *S*. Schwarzengrund isolates have been reported to show antimicrobial resistance and have been genetically characterized [7]. The antimicrobial susceptibility of the remaining 101 *Salmonella* isolates is reported in this study. The same antimicrobial resistance gene combinations in *Salmonella* isolates were often observed in several serovars. However, it remains unknown whether they carry a common antimicrobial resistance plasmid. To confirm that different *S. enterica* serovars carry a common antimicrobial resistance plasmid, antimicrobial resistance transfer was examined in selected *S.* Infantis and *S.* Schwarzengrund isolates, the major serovars obtained from chicken meat. Whole-genome sequencing (WGS) was performed on representative isolates.

## 2. Results

### 2.1. Antimicrobial Susceptibilities of the Isolates and Their Antimicrobial Resistance Genes

Table 1 shows the 16 antimicrobial resistance gene patterns detected via PCR among the 101 *Salmonella* isolates investigated in this study. The most predominant pattern was *aac(6′)-Iaa*, *aadA1*, *tetA*, *sul1*, and *dfrA14*, as confirmed in 32 *S*. Infantis isolates, followed by *aac(6′)-Iaa*, *aadA1*, *tetA*, *sul1*, *dfrA14*, and *aphA1*, as confirmed in 18 *S*. Infantis isolates. The third most common pattern, *aac(6′)-Iaa*, *aadA1*, *tetA*, and *sul1*, was found in six *S*. Manhattan, five *S*. Infantis, three *S*. Agona, and two *S*. Yovokome isolates.

Antimicrobial resistance was observed depending on the antimicrobial resistance genes detected via PCR. However, *sul1* and *dfrA14* did not completely confer antimicrobial resistance. We also found that 27 *sul1*-positive and 46 *dfrA14*-positive isolates were susceptible to trimethoprim–sulfamethoxazole and trimethoprim, respectively. Eleven isolates (four *S*. Typhimurium, three *S*. Infantis, two *S*. Yovokome, one *S*. Manhattan, and one serovar Untypable [OUT: r: 1,5]) were resistant to nalidixic acid. All 101 isolates were susceptible to ciprofloxacin (minimum inhibitory concentration [MIC], 0.25 to 0.5 μg/mL).

All 80 *aadA*-positive isolates detected via PCR harbored 1.0-kb class 1 integrons (Table 1). Agarose gel electrophoresis of the FastDigest TaqI-digested PCR products for gene cassettes of class 1 integrons showed that the restriction fragment length polymorphism (RFLP) patterns of 78 isolates matched those of the *aadA1*-positive isolate (Sal_G1) sequenced in a previous study [7]. The RFLP patterns of the remaining two *S*. Infantis isolates (Sal_45 and Sal_157) were the same but different from those of the *aadA1*-positive isolate. The amplicons of these two isolates were sequenced, and DNA alignment showed 100% (918/918) identity with the class 1 integron containing the *aadA2* gene cassette (accession no. CP040321; position, 4,110,362 to 4,111,279). The results of the discrimination of the harboring *aadA* detected by PCR into *aadA1* and *aadA2* according to the RFLP patterns are shown in Table 1.

### 2.2. Horizontal Transfer of Antimicrobial Resistance

Table 2 shows the results of the transfer of antimicrobial resistance tests using 29 *Salmonella* isolates as donors. Antimicrobial resistances were transferred from 20 of the 29 donor isolates to the recipient *Escherichia coli* DH5α-R3. The antimicrobial resistance genes detected via PCR in donors and transconjugants are shown in Table 2. Although the antimicrobial resistance gene that the donor harbored was tested via PCR in transconjugants, the *aac(6′)-Iaa* on a chromosome was excluded from testing. All harbored antimicrobial resistance genes were transferred from most donors. Sal_235 yielded transconjugants with four different antimicrobial resistance patterns. Transconjugants were obtained using deoxycholate–hydrogen sulfide–lactose (DHL) agar medium supplemented with streptomycin, tetracycline, or kanamycin. From three donor isolates (Sal_287, Sal_289, and Sal_291) harboring *strA*, *strB*, *sul2*, *dfrA14*, and *aphA1*, only *aphA1* was transferred. Transconjugants that acquired only *aphA1* were then obtained using a DHL agar medium supplemented with kanamycin. Supplementary Table S1 shows the antimicrobial susceptibility of the transconjugants. The transconjugants were resistant to the corresponding antimicrobial agents according to the acquired antimicrobial resistance genes. However, seven transconjugants that acquired *dfrA14* but were susceptible to trimethoprim were also observed. In addition, one transconjugant (TC73) did not acquire *aphA1*, but the MIC of kanamycin for TC73 (32 μg/mL) was higher than those of DH5α-R3 (1 μg/mL). The MICs of kanamycin for the other transconjugants that acquired *aphA1* were higher, with values above 256 μg/mL. No transconjugants were obtained from the six donor isolates (*S*. Infantis, Sal_102; *S*. Schwarzengrund, Sal_83, Sal_145, Sal_266, Sal_290, and Sal_294) that harbored only *aphA1*. Moreover, the transfer of antimicrobial resistance genes could not be confirmed from *S*. Infantis Sal_286, which harbored *aadA1*, *tetA*, *sul1*, *dfrA14* and *bla*_CMY-2_; *S*. Infantis Sal_256, which harbored *aadA1*, *tetA*, *sul1*, *dfrA14* and *aphA1*; and *S*. Infantis Sal_80, which harbored *aadA1*, *tetA*, *sul1*, and *dfrA14*. For *S*. Infantis Sal_80, Sal_102, Sal_256, and Sal_286, and *S*. Schwarzengrund Sal_83, Sal_145, Sal_266, Sal_290, and Sal_294 as donors, conjugation experiments were carried out using the broth mating method and/or the filter mating method, with one to six strains of rifampicin-resistant mutants selected from six antimicrobial-susceptible *E. coli* as recipients. The transfer of antimicrobial resistance was not observed in the donor–recipient combinations.

### 2.3. WGS

The number of reads obtained from the short- and long-read sequencing performed in this study is shown in Supplementary Table S2. Short-read sequences for the five *S*. Schwarzengrund isolates, as previously reported [7], were also analyzed in this study, as shown in Supplementary Table S2.

Short-read sequencing was performed on two *S*. Infantis isolates (Sal_238 and Sal_256). The two *S*. Infantis isolates were assigned to ST32 via multilocus sequence-typing (MLST) analysis and core-genome sequence-type (cgST) 40031 or cgST18978 via core-genome multilocus sequence-type (cgMLST) analysis [7,8,9] (Table 3). Six antimicrobial resistance genes and chromosomal point mutation in *parC* (T57S) were detected using the ResFinder (Table 3). IncFIB for Sal_238 and Sal_256 and IncX4 for Sal_256 were determined using PlasmidFinder (Table 3).

Although our previous study reported the presence of antimicrobial resistance genes [7] in five *S*. Schwarzengrund isolates, a chromosomal point mutation in *parC* (T57S) was also confirmed in this study. PlasmidFinder identified an IncFIB plasmid in five *S*. Schwarzengrund isolates (Table 3).

Long-read sequencing was performed on *S*. Infantis Sal_238 and *S*. Schwarzengrund Sal_249. The hybrid assembly yielded contigs of the chromosomes (4,680,940 and 4,659,199 bp) and a circular sequence of one plasmid (288,159 and 288,207 bp) for Sal_238 and Sal_249, respectively. The plasmid sequences in Sal_238 and Sal_249 were registered in the DDBJ database as pSal_238Inf (accession no. LC 785392) and pSal_249Sch (accession no. LC785393), respectively. A comparison of the plasmid sequences of the two isolates showed 99.58% (287,568/288,780 bases) sequence identity. The pSal_238Inf and pSal_249Sch sequences were compared with sequences from the international nucleotide sequence database using BLAST (GENETYX version 15). Although no plasmids matched the full-length sequences of these plasmids, their sequences were highly homologous to those of pESI (CP047882) and pN17S0349 (CP052814). The sequences of pSal_238Inf and pSal_249Sch were 91.94% (274,576/298,632 bases) and 91.93% (274,578/298,679 bases) identical to the pESI sequence, respectively. Thus, pSal_238Inf and pSal_249Sch were identified as pESI-like plasmids. Similarly, the sequences of pSal_238Inf and pSal_249Sch were 82.59% (264,241/319,935 bases) and 82.58% (264,246/319,979 bases) identical to the pN17S0349 sequence, respectively.

**Figure 1:**
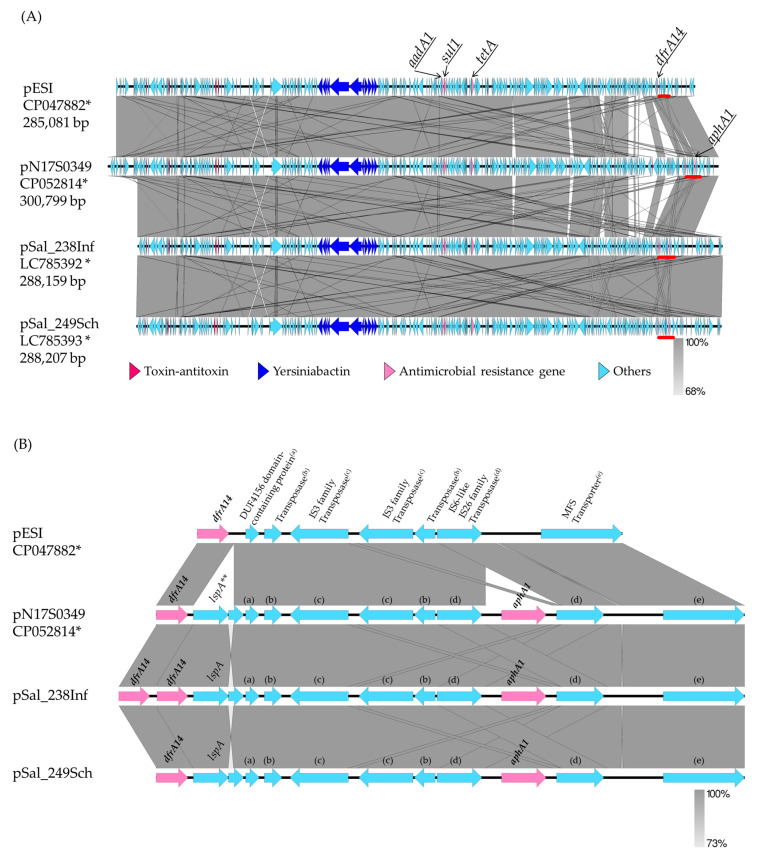
Comparison of plasmid structures among *Salmonella enterica* serovar Infantis and *Salmonella enterica* serovar Schwarzengrund. (**A**) Comparison of the entire plasmid structures of pESI, pN17S0349, and pSal_238Inf in *Salmonella* Infantis isolates obtained in Israel, USA, and Japan, respectively, and pSal_249Sch in *Salmonella* Schwarzengrund obtained in Japan. (**B**) Comparison of gene regions between *dfrA14* and MFS transporter. This figure (**B**) only shows the positions of the following plasmids: pESI, 266456–272779; pN17S0349, 283940–292690; pSal_238Inf, 256081–265387; pSal_249Sch, 256685–265435. The regions marked by the red bars are shown on a larger scale in Figure 1B. * Accession no., ** *lspA*, signal peptidase II; (a) DUF4156 domain-containing protein; (b) transposase; (c) IS3 family transposase; (d) IS6-like IS26 family transposase; (e) MFS transporter.

The sequences of four plasmids (pSal_238Inf, pSal_249Sch, pESI, and pN17S0349) were annotated using the DDBJ Fast Annotation and Submission Tool (DFAST), and the plasmid structures were drawn and compared using Easyfig (Figure 1A). In the region between *dfrA14* and the MFS transporter, the *lspA* and *aphA1* genes were not found in the pESI but were inserted at the same position in pSal_238Inf, pSal_249Sch, and pN17S0349 (Figure 1B). Moreover, two *dfrA14* genes were identified in pSal_238Inf (Figure 1B).

Next, the short-read sequences of *S*. Schwarzengrund Sal_167, Sal_266, Sal_278, and Sal_291 were aligned to the reference pSal_249Sch sequence to obtain each consensus sequence. Short-read sequences of *S*. Infantis Sal_256 were also aligned with the sequence of pSal_238Inf as a reference. Contigs were obtained via de novo assembly using short-read sequences from these five isolates. Contigs with high homology to the consensus sequences of the pSal_249Sch or pSal_238Inf isolates were selected using local BLAST with GENETYX. The number of contigs, coverage, and base match rates for the consensus sequence are shown in Supplementary Table S3. Because the coverage and concordance rates were at least 94.29% and 94.31%, respectively, the consensus sequence was used as the tentative plasmid sequence. The tentative plasmid sequences were then annotated using DFAST, and the plasmid structure was constructed using Easyfig (Supplementary Figure S1). The sequences of pSal_278 and pSal_167 showed high homology to the pSal_249Sch sequence [99.07% (285,562/288,216 bases) and 98.71% (284,521/288,215 bases), respectively], whereas the sequences of pSal_291 and pSal_266 were less identical [88.78% (255,898/288,214 bases) and 77.97% (224,729/288,218 bases), respectively] to the pSal_249Sch sequence. Similarly, the sequences of pSal_256 showed high homology with those of pSal_238Inf [99.21% (285,921/288,170 bases)].

Two plasmid sequences (pSal_238Inf and pSal_249Sch) and five tentative plasmid sequences (pSal_278, pSal_167, pSal_291, pSal_266, and pSal_256) contained the yersiniabactin biosynthetic, regulatory, and transport operons and the toxin–antitoxin system (Figure 1A and Figure S1). The presence of yersiniabactin siderophore genes, which are characteristic of pESI, was tested for all isolates using PCR targeting the *irp2* gene [9]. All *S*. Schwarzengrund, *S.* Infantis, *S*. Manhattan, *S*. Agona, and *S*. Yovokome isolates and one serovar UT (OUT: r: 1,5) isolate were positive for *irp2* (Table 4). Four of the ten *S*. Typhimurium isolates were also positive for *irp2*; one *S*. Kedougou isolate was negative for *irp2* (Table 4).

Although the presence of *acc(6′)-Iaa* was confirmed on the chromosome, the remaining antimicrobial resistance genes confirmed on two plasmid sequences and five tentative plasmid sequences matched the genes confirmed to be harbored by those isolates, as determined using PCR (Figure 1A and Table 2). As an exception, *strA*, *strB*, and *sul2*, which were detected via PCR and ResFinder for Sal_291, were not on the tentative pSal_291 sequence, as pSal_249Sch, which was used as a reference, did not contain them.

All seven isolates for which the plasmid structures were determined or tentatively determined were tested for antimicrobial resistance transfer (Table 2). The determined or tentatively determined plasmid structures were then compared to evaluate antimicrobial resistance transfer. No transfer of antimicrobial resistance was observed for Sal_266 and Sal_256. The region from position 170,499 to 221,059 in pSal_249Sch included genes involved in plasmid transfer. Although the region from position 149,229 to 203,510 of pSal_249Sch, including part of the transfer region, was not confirmed in pSal_266, the entire transfer region was confirmed in pSal_256, similar to pSal_238Inf (Supplementary Figure S1). For nine isolates, including Sal_266 and Sal_256, which did not transfer antimicrobial resistance (Table 2), the presence of *pilV*, *traU*, and *traW* in the transfer region was tested using PCR. Moreover, 32 *S*. Schwarzengrund isolates and two *S*. Infantis isolates that were not tested for antimicrobial resistance transfer were tested for *pilV*, *traU*, and *traW*. Three isolates that exhibited the transfer of antimicrobial resistance (Sal_287, Sal_289, and Sal_291) were used as positive controls. Sal_266, which contained *traW* but not *pilV* or *traU* on the tentative plasmid, was also positive for *traW* and negative for *pilV* and *traU* via PCR. Sal_256 and Sal_291, which contain three genes on each tentative plasmid, were also positive for all of the *pilV*, *traU*, and *traW* genes. Eight isolates with no confirmed antimicrobial resistance transfer, other than Sal_266, tested positive for these three genes using PCR. Of the 34 isolates not tested for antimicrobial resistance transfer, one isolate (Sal_57) was negative for *pilV* only, and one isolate (Sal_G68) was negative for both *pilV* and *traU*.

Although one *dfrA14* was detected on pSal_249Sch, pESI, and pN17S0349, two *dfrA14* positioned side by side were detected on pSal_238Inf (Figure 1A,B). Therefore, a PCR was carried out on the other *dfrA14*-positive isolates to test whether they contained two *dfrA14* genes adjacent to each other. The primers used here were the same as those used for *dfrA14* detection. The extension time for amplification was increased to 90 s, and the number of cycles was increased to 40. Three *S*. Infantis isolates, from which a 1,021-bp product was amplified, were presumed to be two sequential *dfrA14* genes as Sal_238. The three *S*. Infantis isolates and Sal_238 were obtained from chicken meat in the same prefecture. The remaining 14 *S*. Infantis isolates from chicken meat from the same prefecture, as well as *S*. Infantis and *S*. Schwarzengrund isolates from chicken meat produced in other prefectures, harbored one *dfrA14* gene that yielded an amplification product that is 455 bp long.

### 2.4. cgMLST Analysis for S. Schwaruzengrund Isolates Carrying pESI-Like Plasmids in Japan, the UK, and the USA

**Figure 2:**
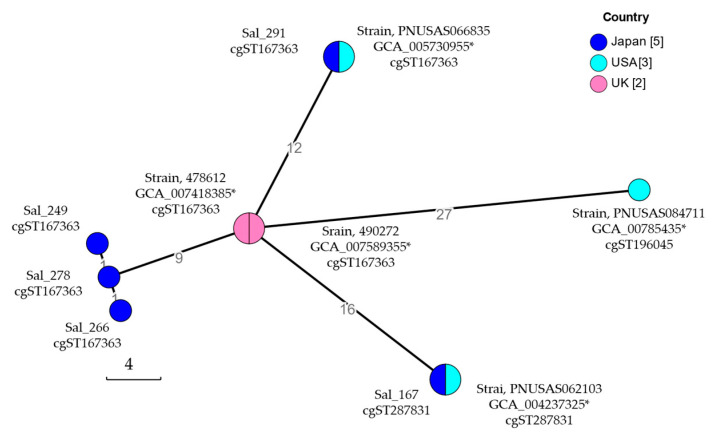
Minimum spanning tree of core-genome multi-locus sequence typing (cgMLST) of *Salmonella enterica* serovar Schwaruzengrund carrying pESI-like plasmids. cgMLST analysis was performed through cgMLSTFinder [8]. The core genomic relationships of *Salmonella* Schwaruzengrund isolates obtained in Japan, the UK, and the USA were visualized using the algorithms of MSTree V2 with GrapeTree software version 1.1. Nodes are color-coded according to the country where each isolate was obtained. The isolate ID or assembly no. and the assigned core-genome sequence type (cgST) of the isolate included in that node are listed near the node. The number of isolates in each country analyzed is shown in square parentheses. * Assembly No.

A cgMLST analysis was performed for five *S.* Schwarzengrund isolates from Japan and the genomes of *S*. Schwarzengrund strains carrying a pESI-like plasmid [8]; strains 478612 (Assembly No. GCA_007418385) and 490272 (GCA_007589355) obtained from clinical samples from the UK; and PNUSAS062103 (GCA_004237325), PNUSAS066835 (GCA_005730955), and PNUSAS084711 (GCA_007854735) obtained from clinical samples from the USA. The assigned cgSTs are listed in Table 3. The major cgST gene is cgST167363. A phylogenetic tree based on cgMLST allele data of these *S.* Schwarzengrund isolates was then visualized using MSTree V2 algorithms with GrapeTree software. A minimum spanning tree based on the cgMLST data is shown in Figure 2. The minimum spanning tree branched out from a node that included only two UK strains, whereas the Japanese and USA strains were classified into six nodes. Each node included an isolate from Japan and a strain from the USA. All five isolates from Japan were included in different nodes.

## 3. Discussion

*S.* Infantis is one of the most commonly isolated strains from human patients and chickens and has recently emerged worldwide [4,10,11,12,13]. In Israel, *S*. Infantis emerged rapidly in 2006–2007; the emerging *S*. Infantis carried a mosaic megaplasmid (~280 kb). The resulting plasmid was named pESI [5]. Comparative analyses between pre-emergent and emergent *S*. Infantis suggested that pESI played an important role in the spread of *S*. Infantis in Israel for 2–3 years [5]. The increase of *S*. Infantis among broiler chickens and human patients worldwide has also been associated with a pESI-like plasmid [4].

In 2018, *S*. Muenchen showed an increase in Israel. Then, in 2019–2020, *S*. Muenchen was the dominant serovar isolated from clinical, poultry, and food sources in Israel. The emerging *S*. Muenchen carried a pESI-like plasmid with a 99.96% nucleotide sequence identity to the pESI carried by *S*. Infantis. This is the second reported case of pathogen emergence and spread associated with pESI-like plasmid acquisition [14]. We previously reported that *S*. Schwarzengrund ST241 expanded its distribution from Western to Eastern Japan [7]. The present study revealed that antimicrobial-resistant *S*. Schwarzengrund, which has expanded its distribution, carries a pESI-like plasmid. PCR targeting the *irp2* gene suggested that, in 2008, when the distribution of *S*. Schwarzengrund in Japan was restricted to Western Japan [15], it already carried a pESI-like plasmid. Although previous bioinformatics analyses of *Salmonella* genomes revealed a pESI-like plasmid structure in *S*. Schwarzengrund in the UK and USA [8], to our knowledge, there are no reports on the distribution of *S*. Schwarzengrund strains with pESI-like plasmids. The present study reported that *S*. Schwarzengrund was the third serovar of *Salmonella* to expand, whose distribution expanded in association with pESI-like plasmid acquisition in Japan.

*S*. Infantis carrying pESI emerged rapidly in Israel in 2006–2007 [5]. The prevalence of *S*. Muenchen carrying pESI also increased in 2018 and rapidly became the most predominant serovar isolated in Israel in 2019–2020 [14]. The present study revealed that *S*. Schwarzengrund already carried a pESI-like plasmid in 2008. However, seven years later, in 2015, its distribution was reported to have expanded to the entire country for the first time, with an isolation rate of 23.3% (56/240), lower than that of *S*. Infantis (77/240, 32.1%) [6]. From 2018 to 2021, *S*. Schwarzengrund was the most commonly isolated serovar from chicken meat products collected in Japan (146/235, 62.1%), followed by *S*. Infantis (30/235, 12.8%) [16]. The expansion of the distribution of *S*. Schwarzengrund containing pESI-like plasmids and the increase in its contamination rate did not occur as rapidly as those of *S*. Infantis [5] and *S*. Muenchen [14] in Israel. Although *S*. Typhimurium (4/10), *S*. Manhattan (7/7), *S*. Agona (3/3), and *S*. Yovokome (2/2) obtained from chicken meat collected between 2015 and 2017 were also suggested to carry a pESI-like plasmid following the results of *irp2*-targeted PCR, the isolation percentage of these serovars did not increase in Japan from 2018 to 2021 [16]. There may be differences in the changes of the characteristics of pathogens by pESI depending on the serovar, such as increasing the ability of *S.* Infantis to spread infection. The presence of pESI-like plasmids in *S*. Agona isolated in Japan in 2014 has already been reported [8]. However, to the best of our knowledge, there are no reports of *S*. Manhattan and *S*. Yovokome carrying a pESI-like plasmid. In a previous study using a mouse model, following *S*. Infantis infection, pESI was horizontally transferred to the gut microbiota and then to *S*. Typhimurium [17]. However, there have been no reports of *S*. Typhimurium pESI-carrying isolates from clinical, animal, or food samples.

By performing a genomic analysis of *Salmonella* genomes from the National Center for Biotechnology Information (NCBI), the structure of the pESI-like plasmid was confirmed in five *S.* Schwarzengrund isolates obtained from clinical samples in the UK in 2018 and in the USA (isolation date unknown) [8]. All five *S*. Schwarzengrund isolates with pESI-like plasmids from the UK and USA were classified as ST241 via MLST [8]. The five *S*. Schwarzengrund isolates from Japan were confirmed to carry pESI-like plasmids via genome analysis in this study and were assigned to the same ST, ST241 [7]. The cgMLST performed in this study revealed that 10 *S*. Schwarzengrund isolates with a pESI-like plasmid were classified into three cgSTs that were closely related. However, differences were identified in their core genomes. From the minimum spanning tree obtained using cgMLST, all five isolates from Japan were classified into different nodes, indicating that they acquired more genetic variation than the two UK strains classified in the same node. In Japan, *S*. Schwarzengrund has been isolated from broiler [18,19] and chicken meat [6,15], and the number of imported and exported chickens was confirmed through animal quarantine statistics [20]. Japan exported an average of 6,954 tons of poultry meat annually between 2019 and 2022, with yearly exports ranging from 3289 to 10,016 tons. During this period, no live chickens were exported. The export destinations for poultry meat from Japan have not been published. However, the amount of exported poultry meat is not large compared to the amount of chicken meat and number of live chickens that Japan imported. Japan is an established importer of one-day-old chicks. In 2014, 37.1% (170,245/459,443) of the one-day-old chicks imported to Japan came from the UK and 7.6% (34,825/459,443) from the USA. In 2019, the percentage of one-day-old chicks imported from the UK and the USA increased to 60.5% (186,563/308,592) and 11.4% (35,182/308,592), respectively. The predominant serovars of *Salmonella* isolated from retail chickens in the USA during 2013–2020 were *S*. Enteritidis, *S*. Infantis, *S*. Kentucky, and *S*. Typhimurium; *S*. Schwarzengrund was neither isolated nor considered a minor serovar [21]. Among *Salmonella* isolates from foodborne outbreaks in the USA from 1998 to 2021, the most predominant serovar was *S*. Enteritidis (28.42%); *S*. Schwarzengrund (0.52%) was a minor serovar [22]. In the UK, only *S*. Infantis (3/310) and *S*. Java (2/310) were detected in samples of frozen, raw, or partly cooked coated chicken products collected from retailers in 2021 [23]. These data suggest that *S*. Schwarzengrund was a minor serovar in the UK and USA, and no evidence of pESI-carrying *S*. Schwarzengrund entering Japan from both countries was found.

Over the last 10 years, multidrug-resistant *S*. Infantis has spread globally. Specifically, *S.* Infantis isolates in recent years have been reported to carry a pESI-like plasmid [4]. In Japan, *S*. Infantis was the predominant serovar in *Salmonella*-contaminated chicken meat from 1995 to 1998. The antimicrobial resistance and plasmids of these *S*. Infantis isolates have not been examined [24]. *S*. Infantis isolates from chickens in Japan in 2011 were subjected to PCR targeting the *ipr2* gene. Consequently, *S*. Infantis isolates positive for the *ipr2* gene were considered to carry a pESI-like plasmid [25]. In the present study, complete plasmid sequences were identified for *S*. Infantis Sal_238 and *S*. Schwarzengrund Sal_249, which were isolated from chicken meat in Japan in 2016. The high homology between pSal_249Sch and pSal_238Inf suggests that their ancestors were common. pSal_249Sch and pSal_238Inf showed high homology to pESI in *S*. Infantis [5] but differed from pSal_249Sch and pSal_238Inf carrying *aphA1*. The pN17S0349 (accession no. CP052814) in *S*. Infantis CVM N17S349 isolated from a ground turkey was identical to pSal_249Sch and pSal_238Inf carrying *aphA1*, but the homology of pN17S0349 to pSal_249Sch and pSal_238Inf was lower than that of pESI. The structure of pESI-like plasmids varies. Nonetheless, antimicrobial resistance genes were inserted into pESI as follows: *aph(3′)-Ia* (*aphA1*), *bla*_CTX-M-65_, *floR*, and *fosA3* in the USA [26]; *bla*_CTX-M-1_, *bla*_CTX-M-65_, *fosA*, and *floR* in Italy [10]; and *bla*_CTX-M-14_ in Russia [27]. In contrast, high homology was observed between pSal_249Sch and pSal_238Inf. Their genetic characteristics suggest the horizontal transfer of a pESI-like plasmid from *S*. Infantis, which was previously widely distributed in Japan, to *S*. Schwarzengrund, which was once a rare serovar in Japan [24,28].

The tentative plasmid sequences of four *S*. Schwarzengrund isolates with different antimicrobial resistance gene patterns and one *S*. Infantis isolate were also determined in this study. Although their plasmid structures were diverse, the tentative plasmid sequences contained K88-like and *Ipf* fimbria; yersiniabactin biosynthetic, regulatory, and transport operons; and type II toxin–antitoxin systems identified on pESI [5]. The type II toxin–antitoxin system found in pESI was suggested to increase the stability of pESI in the host [5]. This suggests that these pESI-like plasmids, which contained the toxin–antitoxin system, were also stably maintained in *S*. Schwarzengrund and *S*. Infantis in Japan. The structure of the pESI-like plasmid carried by *S*. Schwarzengrund in Japan changed with the deletion of the plasmid transfer region (pSal_266 and pSal_291) or class 1 integrons, antimicrobial resistance genes (*aadA1*, *sul1*, and *tetA*), and the mercury resistance operon (pSal_266 and pSal_291). Although *Salmonella* carrying pESI-like plasmids lacking these genes would not be able to increase under antimicrobial selection pressure, the percentage of isolates harbored *aadA1*, *sul1*, and *tetA* significantly decreased from 2008 to 2015–2019 [7]. The prudent use of antimicrobials is widely practiced, and *Salmonella* that have lost these genes may be able to continue to survive in chicken flocks. The diversity in the structure of pESI-like plasmids suggests that some time had passed after *S*. Schwarzengrund acquired the pESI-like plasmid, not immediately.

*Salmonella* carrying pESI-like plasmids in Japan typically contained *aac(6′)-Iaa*, *aadA1*, *sul1*, *tetA*, and *dfrA14*. In addition, many isolates harbored *aphA1*. Based on the characteristics of the antimicrobial resistance gene pattern, we hypothesized that different serovars among *Salmonella* isolates shared a common plasmid. However, pESI-like plasmids were detected via PCR even if *S*. Schwarzengrund, *S*. Infantis, and other isolates harbored only *aac(6′)-Iaa* or only one or two additional antimicrobial resistance genes. Therefore, the presence of a pESI-like plasmid should not be assumed based on the combination of antimicrobial resistance genes alone. Furthermore, pESI was reported to be a self-transmissible plasmid [5]. However, deletion of the plasmid transfer region in a pESI-like plasmid was confirmed. The plasmid lost its ability to be transferred. This suggested that the transfer region was deleted after the pESI-like plasmid was transferred between the *Salmonella* isolates from different serovars.

## 4. Materials and Methods

### 4.1. Salmonella Isolates

From the *Salmonella* isolates obtained from chicken meat in a previous report [6], 101 isolates, excluding *S*. Schwarzengrund, were tested for antimicrobial susceptibility in this study, including *Salmonella* Infantis (77 isolates), *S*. Typhimurium (10 isolates), *S*. Manhattan (seven isolates), *S*. Agona (three isolates), *S*. Yovokome (two isolates), *S*. Kedougou (one isolate), and an Untypable isolate (O Untypable [UT]: r: 1,5) (one isolate). Although the antimicrobial resistances of 124 *S*. Schwarzengrund isolates obtained from chicken meat collected in 2008 and between 2015 and 2019 were reported in our previous study [7], these *S*. Schwarzengrund isolates were used in this study for analyses other than antimicrobial resistance.

### 4.2. Antimicrobial Susceptibility Tests

The minimum inhibitory concentrations (MICs) of nine antimicrobial agents [ampicillin (breakpoint, 32 μg/mL), cefazolin (8 μg/mL), gentamicin (16 μg/mL), kanamycin (64 μg/mL), tetracycline (16 μg/mL), chloramphenicol (32 μg/mL), nalidixic acid (32 μg/mL), ciprofloxacin (1 μg/mL), and trimethoprim–sulfamethoxazole (4/76 μg/mL)] on the 101 investigated isolates were determined with the broth microdilution method using Frozen Plate (Eiken Chemical Co., Ltd., Tokyo, Japan). In addition, the MICs for streptomycin (16 μg/mL) (FUJIFILM Wako Pure Chemical Co., Osaka, Japan) and trimethoprim (16 μg/mL) (FUJIFILM Wako Pure Chemical Co.) were determined using the agar dilution method in Mueller–Hinton agar (Thermo Fisher Scientific K. K., Osaka, Japan), according to the Clinical Laboratory Standards Institute guidelines [29].

### 4.3. Antimicrobial Resistance Gene and Integron Detection

The major genes for resistance to streptomycin [*aac(6′)-Iaa*, *aadA* (another name, *ant(3″)-Ia*) and *strA* (*aph(3″-Ib*)), *strB* (*aph(6)-Id*)], kanamycin [*aphA1* (*aph(3′)-Ia*), and *aphA2* (*aph(3′)-IIa*)], tetracycline (*tetA* and *tetB*), sulfamethoxazole (*sul1* and *sul2*), and trimethoprim (*dfrA14*) were tested using simplex or multiplex PCR, as reported previously [7,30]. Moreover, the genes conferring resistance to ampicillin (*bla*_TEM_) [30] and cefazolin (*bla*_CTX-M_ [31] and *bla*_CMY-2_ [30] were tested using multiplex PCR.

To determine the inserted gene cassettes in the class 1 integron, the corresponding regions in each isolate were classified using RFLP analysis, as previously described [7,32]. The primer sequences used for PCR in this step are listed in Supplementary Table S4.

### 4.4. Conjugation Assay

Next, conjugation experiments were performed using the broth mating method to determine the transfer of antimicrobial resistance genes. A rifampicin-resistant *E. coli* strain DH5α-3R was used as the recipient strain [33]. Briefly, the donor and recipient strains were each inoculated into 3 mL of Mueller–Hinton broth (Thermo Fisher Scientific K. K.) and pre-cultured for 6 h at 35 °C. One milliliter of the recipient strain culture was centrifuged at 12,000 rpm for 3 min, and the recipient cells were resuspended in 3 mL of fresh Mueller–Hinton broth. Next, 0.1 mL of the donor culture medium was inoculated into this recipient culture and co-cultured overnight at 35 °C. Subsequently, 1 mL of the resulting co-culture centrifuged at 12,000 rpm for 3 min was suspended in 100 µL of sterile saline, inoculated onto Mueller–Hinton or DHL agar (Shimadzu Diagnostics Corporation, Tokyo, Japan) supplemented with rifampicin (100 μg/mL) and either streptomycin (5 μg/mL), tetracycline (12.5 μg/mL) (FUJIFILM Wako Pure Chemical Co.), or kanamycin (100 μg/mL) (Tokyo Chemical Industry Co., Ltd., Tokyo, Japan), and incubated at 35 °C for 24 h. Up to four colonies of candidate transconjugant strains were selected per donor and subcultured on additive-free Mueller–Hinton agar.

The agar dilution method was used to determine the MICs of ampicillin, cefazolin, streptomycin, kanamycin, tetracycline, and trimethoprim for the transconjugants. The antimicrobial resistance genes in the transconjugants were also detected via PCR, as described above.

### 4.5. WGS Analysis

Short-read sequencing for five *S*. Schwarzengrund isolates (Sal_167, Sal_249, Sal_266, Sal_278, and Sal_291) was performed as in our previous study [7]. In the present study, for representative *S*. Infantis isolates, cDNA libraries were prepared using a Nextera XT Library Prep Kit (Illumina K. K., San Diego, CA, USA) according to the manufacturer’s instructions and sequenced on an iSeq System (Illumina K. K.), as previously reported [7,34]. To accurately characterize the structure of the plasmids, DNA was purified using a FavorPrep tissue genomic DNA extraction mini kit (Chiyoda Science Co., Ltd., Tokyo, Japan) following the manufacturer’s instructions. cDNA libraries were prepared using a Rapid Barcoding Sequencing kit (SQK-RBK001; Oxford Nanopore Technologies, Oxford, UK), and long-read sequencing was performed on a MinION flow cell R9.4 system (Oxford Nanopore Technologies).

Trimming of raw short reads and long-read data was performed using FastP (v 0.23.3) [35] and filtlong v 0.2.1 (https://github.com/rrwick/Filtlong, accessed on 25 May 2023). The long-read sequences obtained using MinION were corrected with short reads obtained via iSeq using LoRDEC [36]. Complete plasmid sequences were constructed via de novo assembly of corrected long-read data using Flye v 2.9.2 [37]. DFAST was used to annotate the genome [38]. The Nucleotide BLAST program was used to compare DNA alignments with data from the NCBI using GENETYX version 15 (Genetyx Corporation, Tokyo, Japan). Multiple alignment using the Fast Fourier Transform (MAFFT) version 7 (https://mafft.cbrc.jp/alignment/software/, accessed on 5 April 2024) was used for nucleotide sequence alignment. The tentative plasmid sequence was also determined from the short-read sequences of the isolates that were not long-sequenced. Short-read sequences were aligned to the determined plasmid sequence as a reference to obtain each consensus sequence with the mapping function using GENETYX-NGS version 5 (Genetyx Corporation). Contigs were also obtained via the de novo assembly of short-read sequences using GENETYX-NGS. Contigs with high homology to the consensus sequences of each isolate were selected using local BLAST with GENETYX. The percentage of tentative plasmid sequences covered by the contig obtained in the de novo assembly and the percentage of nucleotide sequence matches were further confirmed. To compare the plasmid structures, Easyfig was used for map generation [39]. Bacterial genome contigs were scanned against the ResFinder, PointFinder, PlasmidFinder, and PubMLST databases using staramr [40]. Some genes detected by the genome analysis were tested using PCR for other isolates.

Core-genome multilocus sequence-type (cgMLST) analysis was performed using cgMLSTFinder [41,42] for *S.* Schwaruzengrund isolates obtained in Japan (Sal_167, Sal_249, Sal_266, Sal_278, and Sal_291 from chicken meat) and genome data obtained from the NCBI database (https://www.ncbi.nlm.nih.gov/datasets/genome/, accessed on 1 November 2024). Genetic relationships were visualized using MSTree V2 algorithms in GrapeTree software [43].

## 5. Conclusions

In this study, complete plasmid sequences were identified for *S. enterica* serovar Schwarzengrund Sal_249 and *S. enterica* serovar Infantis Sal_238 isolated from chicken meat in Japan in 2016. High homology was observed among pSal_249Sch, pSal_238Inf, and pESI. The distribution, expansion, and contamination rate of *S*. Schwarzengrund with pESI-like plasmids increased more slowly compared with those of *S*. Infantis and *S*. Muenchen in Israel. cgMLST analysis showed that *S*. Schwarzengrund isolates with pESI-like plasmids from Japan, the UK, and the USA were closely related. Moreover, the ancestors of these *S*. Schwarzengrund isolates harboring the pESI-like plasmid are suggested to be common. Genomic analysis of more isolates is required to identify the genetic characteristics of pESI-carrying *S*. Schwarzengrund and the factors contributing to the increase in its prevalence.

## Figures and Tables

**Table 1:** Antimicrobial resistance genes present in *Salmonella* isolates obtained from chicken meats in Japan.

	No. of Isolates	*S*. Infantis			*S*. Typhimurium		*S*. Manhattan		Other	
Antimicrobial Resistance Genes		1 kb ^(a)^		No ^(b)^	1 kb	No	1 kb	No	1 kb	No
		*aadA1* ^(c)^	*aadA2*		*aadA1*		*aadA1*		*aadA1*	
*aac(6′)-Iaa, aadA1, tetA, sul1, dfrA14, aphA1, bla*_TEM_	1	1								
*aac(6′)-Iaa, aadA1, tetA, sul1, aphA2, bla*_TEM_	1				1					
*aac(6′)-Iaa, aadA1, tetA, sul1, dfrA14, aphA1*	18	18								
*aac(6′)-Iaa, aadA1, tetA, sul1, dfrA14, bla*_CMY-2_	3	3								
*aac(6′)-Iaa, aadA1, tetA, sul1, dfrA14, bla*_TEM_	1				1					
*aac(6′)-Iaa, aadA1, tetA, sul1, aphA1*	5	5								
*aac(6′)-Iaa, aadA1, tetA, sul1, dfrA14*	32	32								
*aac(6′)-Iaa, aadA1, tetA, sul1, bla*_TEM_	1				1					
*aac(6′)-Iaa, aadA2, tetA, sul1, bla*_CTX-M_	2		2							
*aac(6′)-Iaa, aadA1, tetA, sul1*	16	5					6		5 ^(d)^	
*aac(6′)-Iaa, dfrA14, aphA1, bla*_TEM_	1					1				
*aac(6′)-Iaa, aphA2, bla*_TEM_	6					6				
*aac(6′)-Iaa, dfrA14, aphA1*	5			4						1 ^(e)^
*aac(6′)-Iaa, aphA1*	1			1						
*aac(6′)-Iaa, dfrA14*	5			5						
*aac(6′)-Iaa*	3			1				1		1 ^(f)^
total	101	64	2	11	3	7	6	1	5	2

^(a)^ The length of the class 1 integron; ^(b)^ the absence of class 1 integrons; ^(c)^ antimicrobial resistance genes located on class 1 integron. ^(d)^ three *S*. Agona isolates and two *S*. Yovokome; ^(e)^ O untypable: r: 1,5; ^(f)^ *S*. Kedougou.

**Table 2:** Antimicrobial resistance genes transferred from *Salmonella* Schwarzengrund and *Salmonella* Infantis obtained from chicken in Japan.

Serovar	Donar		Transconjugant
	Isolate ID	Antimicrobial Resistance Genes	Antimicrobial Resistance Genes
*S.* Infantis	Sal_235	*aadA1, tetA, sul1, dfrA14, aphA1, bla*_TEM_	*aadA* **, tetA, sul1, dfrA14, aphA1, bla_TEM_
			*aadA, tetA, sul1, dfrA14, aphA1*
			*aadA, sul1*
			*aadA, bla*_TEM_
*S*. Infantis	Sal_286	*aadA1, tetA, sul1, dfrA14, bla*_CMY-2_	No transfer
*S*. Infantis	Sal_31	*aadA1, tetA, sul1, dfrA14, aphA1*	*aadA, tetA, sul1, dfrA14, aphA1*
*S.* Infantis	Sal_238 *	*aadA1, tetA, sul1, dfrA14, aphA1*	*aadA, tetA, sul1, dfrA14, aphA1*
*S.* Infantis	Sal_180	*aadA1, tetA, sul1, dfrA14, aphA1*	*aadA, tetA, sul1, dfrA14, aphA1*
*S.* Infantis	Sal_181	*aadA1, tetA, sul1, dfrA14, aphA1*	*aadA, tetA, sul1, dfrA14, aphA1*
*S.* Infantis	Sal_256 *	*aadA1, tetA, sul1, dfrA14, aphA1*	No transfer
*S*. Schwarzengrund	Sal_63	*aadA1, tetA, sul1, dfrA14, aphA1*	*aadA, tetA, sul1, dfrA14, aphA1*
*S.* Schwarzengrund	Sal_15	*aadA1, tetA, sul1, dfrA14, aphA1*	*aadA, tetA, sul1, dfrA14, aphA1*
*S.* Schwarzengrund	Sal_249 *	*aadA1, tetA, sul1, dfrA14, aphA1*	*aadA, tetA, sul1, dfrA14, aphA1*
*S.* Schwarzengrund	Sal_278 *	*aadA1, tetA, sul1, dfrA14, aphA1*	*aadA, tetA, sul1, dfrA14, aphA1*
*S.* Infantis	Sal_51	*aadA1, tetA, sul1, aphA1*	*aadA, tetA, sul1, aphA1*
*S*. Schwarzengrund	Sal_272	*aadA1, tetA, sul1, aphA1*	*aadA, tetA, sul1, aphA1*
*S*. Infantis	Sal_80	*aadA1, tetA, sul1, dfrA14*	No transfer
*S*. Infantis	Sal_25	*aadA1, tetA, sul1, dfrA14*	*aadA, tetA, sul1, dfrA14*
*S*. Schwarzengrund	Sal_159	*aadA1, tetA, sul1, dfrA14*	*aadA, tetA, sul1, dfrA14*
*S*. Schwarzengrund	Sal_167 *	*aadA1, tetA, sul1, dfrA14*	*aadA, tetA, sul1, dfrA14*
*S*. Infantis	Sal_157	*aadA2, tetA, sul1, bla*_CTX-M_	*aadA, tetA, sul1, bla*_CTX-M_
*S*. Infantis	Sal_36	*aadA1, tetA, sul1*	*aadA, tetA, sul1*
*S.* Schwarzengrund	Sal_82	*aadA1, tetA, sul1*	*aadA, tetA, sul1*
*S.* Schwarzengrund	Sal_287	*strA, strB, sul2, dfrA14, aphA1*	*aphA1*
	Sal_289	*strA, strB, sul2, dfrA14, aphA1*	*aphA1*
	Sal_291 *	*strA, strB, sul2, dfrA14, aphA1*	*aphA1*
*S*. Infantis	Sal_102	*aphA1*	No transfer
*S*. Schwarzengrund	Five isolates ^†^	*aphA1*	No transfer

* Whole-genome sequencing was performed. ** The distinction between *aadA1* and *aadA2* acquired by the transconjugant was not determined. But, it is assumed that the transferred gene corresponds to *aadA1* or *aadA2* present in the donor. ^†^ Sal_83, Sal_145, Sal_266, Sal_290, and Sal_294.

**Table 3:** Genetic characteristics of *Salmonella* harboring pESI-like plasmid in Japan, the UK, and the USA.

Serovar	Country	Isolation of Year	Isolate ID	ST	cgST	Antimicrobial Resistance Genes *	Chromosomal Point Mutation	Plasmid Replicons	Reference
*S*. Schwarzengrund									
	Japan								
		2016	Sal_167	241 **	287831	*aadA1, sul1, tetA, dfrA14* **	*parC* (T57S)	IncFIB	[7]
		2016	Sal_249	241 **	167363	aadA1, sul1, tetA, dfrA14, aphA1 **	*parC* (T57S)	IncFIB	[7]
		2017	Sal_266	241 **	167363	aphA1 **	*parC* (T57S)	IncFIB	[7]
		2017	Sal_278	241 **	167363	aadA1, sul1, tetA, dfrA14, aphA1 **	*parC* (T57S)	IncFIB	[7]
		2019	Sal_291	241 **	167363	aphA1, strA/strB, dfrA14, sul2 **	*parC* (T57S)	IncFIB	[7]
	USA								
		No data	GCA_004237325	241 **	287831	aadA1, sul1, tetA, dfrA14, aphA1 **	*parC* (T57S) **	IncFIB **	[8]
		No data	GCA_005730955	241 **	167363	Not detected**	*parC* (T57S) **	IncFIB **	[8]
		No data	GCA_007854735	241 **	196045	aadA1, sul1, tetA, dfrA14, aphA1 **	*parC* (T57S) **	IncFIB **	[8]
	UK								
		2018	GCA_007418385	241 **	167363	aadA1, sul1, tetA, dfrA14, aphA1 **	*parC* (T57S) **	IncFIB **	[8]
		2018	GCA_007589355	241 **	167363	*aadA1, sul1, tetA, aphA1* **	*parC* (T57S) **	IncFIB, IncI1-I **	[8]
*S*. Infantis									
	Japan								
		2016	Sal_238	32	40031	*aadA1, sul1, tetA, dfrA14, aphA1*	*parC* (T57S)	IncFIB	[6]
		2016	Sal_256	32	18978	*aadA1, sul1, tetA, dfrA14, aphA1*	*parC* (T57S)	IncFIB, IncX4	[6]

ST, sequence type by multilocus sequence type analysis; cgST, core-genome sequence type by core-genome multilocus sequence-type analysis. * Antimicrobial resistance genes were detected through resFinder. The gene of *aadA1*, *strB* and *aphA1* are aliases for *ant(3′)-Ia*, *aph(6)-Id* and *aph(3′)-Ia*, respectively. The names of the former genes, the same as previously reported [7], are listed in this table. ** These data were already reported in the reference [7].

**Table 4:** Percentage of pESI-positive *Salmonella* isolates in each serovar estimated by *irp2* detected by PCR.

Serovar	Year of Isolation	No. of Isolates		(%)
		Tested	Positive	
*S*. Schwarzengrund	2008	37	37	(100%)
	2015–2019	87	87	(100%)
*S*. Infantis	2015–2017	77	77	(100%)
*S*. Typhimurium	2015–2016	10	4	(40%)
*S*. Manhattan	2016	7	7	(100%)
*S*. Agona	2015–2017	3	3	(100%)
*S*. Yovokome	2015	2	2	(100%)
S. Kedougou	2016	1	0	
OUT: r: 1,5	2016	1	1	(100%)
Total		225	218	(96.9%)

UT, untypable.

## Data Availability

The data presented in this study are available upon request from the corresponding author.

## Supplementary Materials

The following supporting information can be downloaded at https://www.mdpi.com/article/10.3390/antibiotics14030288/s1: Figure S1: Tentative plasmid structure of *Salmonella enterica* serovar Schwarzengrund and serovar Infantis obtained from chicken meat in Japan; Table S1: Antimicrobial susceptibilities and antimicrobial resistance genes for transconjugants; Table S2: Number of reads obtained in short- and long-read sequencing for *Salmonella* isolates from chicken in Japan; Table S3: Summary of tentative plasmid sequences for *Salmonella* Schwarzengrund and *Salmonella* Infantis obtained from chicken in Japan; Table S4: PCR conditions for detecting antimicrobial resistance genes, class 1 integrons, and virulence and transfer-related genes in pESI.

## Abbreviations

The following abbreviations are used in this manuscript:

DFAST	DDBJ Fast Annotation and Submission Tool
MAFFT	Multiple Alignment using Fast Fourier Transform
MIC	Minimum inhibitory concentration
NCBI	National Center for Biotechnology Information
RFLP	Restriction fragment length polymorphism
WGS	Whole-genome sequencing
