# Supplementary material for: Determination of Antimicrobial Resistance Megaplasmid-Like pESI Structures Contributing to the Spread of Salmonella Schwarzengrund in Japan
Source: Antibiotics (Basel). 2025 Mar 10;14(3):288. doi: 10.3390/antibiotics14030288 (PMC11939482; doi:10.3390/antibiotics14030288)
Supplement: Supplementary file 1 [file antibiotics-14-00288-s001.zip › antibiotics-3516295-supplementary.pdf]

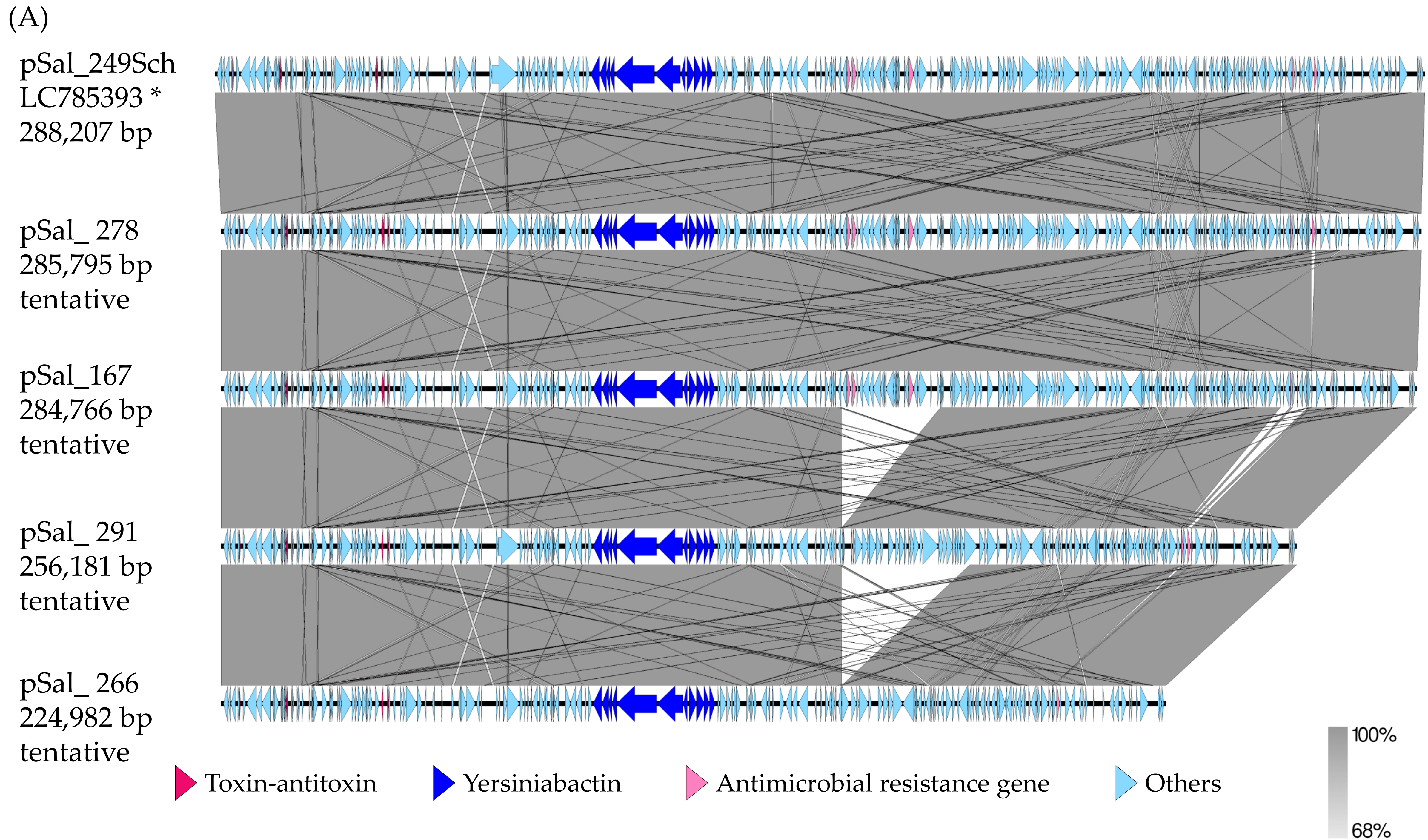
(A)
pSal_249Sch
LC785393 *
288,207 bp
pSal_ 278
285,795 bp
tentative
pSal_167
284,766 bp
tentative
pSal_ 291
256,181 bp
tentative
pSal_ 266
224,982 bp
tentative
Toxin-antitoxin
Yersiniabactin
Antimicrobial resistance gene
Others
100%
68%

(B)

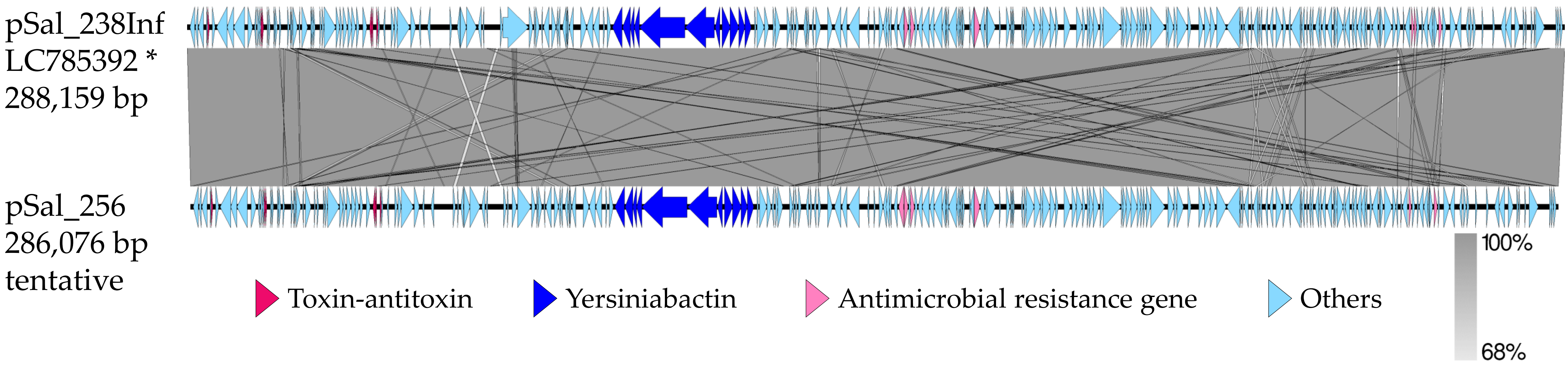

Supplementary Figure S1. Tentative plasmid structure of *Salmonella enterica* serovar Schwarzengrund and serovar Infantis obtained from chicken meat in Japan.
The plasmid structures of pSal_249Sch and pSal_238Inf were determined by hybrid assembly of short- and long-read sequencing. These are the same as in Figure 1, but also shown in this figure as references.
(A) Tentative plasmid structure of *Salmonella enterica* serovar Schwarzengrund:
The plasmid structures of four *Salmonella* Schwarzengrund isolates shown below are tentative, determined by assembly, using the sequence of pSal_249Sch as reference.
(B) Tentative plasmid structure of *Salmonella enterica* serovar Infantis: The tentative pSal_256 was determined in the same way as in (A), using pSal_238Inf as a reference.

Supplementary Table S1. Antimicrobial susceptibilities and antimicrobial resistance genes for transconjugants.

ID		Antimicrobial resistance genes	MICs (μg/ml)					
Donor	Transconjugant		STR	TET	TMP	KAN	AMP	CFZ
Sal_235	TC62	*aadA, tetA, sul1, dfrA14, aphA1, bla* $_{TEM}$	**4**	**32**	**>512**	**256**	**128**	NT
	TC65	*aadA, tetA, sul1, dfrA14, aphA1*	**4**	**32**	**>512**	**256**	1	NT
	TC69	*aadA, sul1*	**8**	1	0.25	1	1	NT
	TC73	*aadA, bla* $_{TEM}$	**16**	1	0.25	**32**	**256**	NT
Sal_286	Not obrained							
Sal_31	TC31	*aadA, tetA, sul1, dfrA14, aphA1*	**16**	**128**	**>512**	**>512**	NT	NT
Sal_238	TC42	*aadA, tetA, sul1, dfrA14, aphA1*	**4**	**16**	**>512**	**256**	NT	NT
Sal_180	TC77	*aadA, tetA, sul1, dfrA14, aphA1*	**4**	**32**	0.5	**256**	NT	NT
Sal_181	TC81	*aadA, tetA, sul1, dfrA14, aphA1*	**4**	**16**	0.5	**256**	NT	NT
Sal_256	Not obrained							
Sal_63	TC44	*aadA, tetA, sul1, dfrA14, aphA1*	**16**	**32**	**1**	**>512**	NT	NT
Sal_15	TC49	*aadA, tetA, sul1, dfrA14, aphA1*	**64**	**16**	0.5	**>512**	NT	NT
Sal_249	TC46	*aadA, tetA, sul1, dfrA14, aphA1*	**8**	**16**	0.5	**512**	NT	NT
Sal_278	TC51	*aadA, tetA, sul1, dfrA14, aphA1*	**4**	**16**	0.5	**256**	NT	NT
Sal_51	TC26	*aadA, tetA, sul1, aphA1*	**4**	**16**	0.25	**256**	NT	NT
Sal_272	TC50	*aadA, tetA, sul1, aphA1*	**32**	**32**	0.5	**512**	NT	NT
Sal_80	Not obrained							
Sal_25	TC57	*aadA, tetA, sul1, dfrA14*	**16**	**16**	**1**	2	NT	NT
Sal_159	TC61	*aadA, tetA, sul1, dfrA14*	**4**	**16**	0.5	1	NT	NT
Sal_167	TC92	*aadA, tetA, sul1, dfrA14*	**4**	**16**	0.5	1	NT	NT
Sal_157	TC90	*aadA2, tetA, sul1, bla* $_{CTX-M}$	**16**	**32**	0.25	1	**>512**	**>512**
Sal_36	TC58	*aadA, tetA, sul1*	**4**	**32**	0.25	1	NT	NT
Sal_82	TC60	*aadA, tetA, sul1*	**16**	**32**	0.25	2	NT	NT
Sal_287	TC85	*aphA1*	<0.5	<0.5	0.25	**256**	NT	NT
Sal_289	TC86	*aphA1*	1	<0.5	0.25	**512**	NT	NT
Sal_291	TC66	*aphA1*	1	1	0.25	**256**	NT	NT
DH5α-R3*			1	1	0.25	1	1	<0.5

*Recipent strain; MICs that are four fold higher than the MIC for the recipient are in bold. MIC values that were not associated with acquired antimicrobial resistance genes and antimicrobial susceptibility are underlined.

** The distinction between *aadA1* and *aadA2* acquired by the transconjugant was not determined. But, it is assumed that the transferred gene corresponds to *aadA1* or *aadA2* present in the donor.

MICs, minimum inhibitory concentrations; STR, streptomycin; TET, tetracycline; TMP, trimethoprim; KAN, kanamycin; AMP, ampicillin; CFZ, cefazolin; NT, not tested.

Supplementary Table S2. Number of reads obtained in short- and long-read sequencing for *Salmonella* isolates from chicken in Japan

Isolate ID	Total reads	
	Short*	Long**
S. Schwarzengrund		
Sal_167	1.79 M	-
Sal_249	1.49 M	37,643
Sal_266	2.07 M	-
Sal_278	1.99 M	-
Sal_291	2.42 M	-
S. Infantis		
Sal_238	2.05 M	24,867
Sal_256	1.36 M	-

-, Not done

*Short-read sequencing

**Long-read sequencing

Supplementary Table S3. Summary of tentative plasmid sequences for *Salmonella* Schwarzengrund and *Salmonella* Infantis obtained from chicken in Japan.

Isolate ID	Plasmid length (bp)[a]	No. of contigs with nucleotide sequence matches to the consensus sequence	Total length of contig (bp)[b]	The rate of cover (%)[c]	No. of matched bases (pb)[d]	The rate of identity (%)[e]
S. Schwarzengrund						
Sal_167	284,776	8	278,258	97.71%	278,031	97.63%
Sal_266	224,982	8	219,387	97.51%	219,170	97.42%
Sal_278	285,795	8	279,402	97.76%	279,187	97.69%
Sal_291	256,181	6	250,771	97.89%	250,493	97.78%
S. Infantis						
Sal_256	286,076	7	269,739	94.29%	269,794	94.31%

a, The length of each tentative plasmid sequence is shown. Each tentative plasmid sequence was determined by aligning the short read sequences to the sequence of pSal_249Sch or pSal_238Inf as a reference.

b, The total length of contigs that matched tentative plasmid sequences by search with LocalBLAST against contigs obtained by *de novo* assembly of short read sequences.

c, The ratio was calculated by dividing (b) by (a).

d, Number of bases matched among the identified contigs by searching the tentative plasmid sequence with LocalBLAST.

e, The ratio was calculated by dividing (d) by (a).

Supplementary Table S4. PCR conditions for detection of antimicrobial resistance genes, class 1 intergron, virulence and transfer-related genes on pESI.

Targeted gene	F/R*	Primer sequences 5' - 3'	Expected products size	Final primer conc.	Annealing Temp.	Reference
Multiplex PCR 1						
aadA	F	GTG GAT GGC GGC CTG AAG CC	525 bp	0.1 µM	63°C	30
	R	AAT GCC CAG TCG GCA GCG				
strA/strB	F	ATG GTG GAC CCT AAA ACT CT	893 bp	0.4 µM		30
	R	CGT CTA GGA TCG AGA CAA AG				
Multiplex PCR 2						
tetA	F	GGC GGT CTT CTT CAT CAT GC	502 bp	0.1 µM	63°C	30
	R	CGG CAG GCA GAG CAA GTA GA				
tetB	F	CGC CCA GTG CTG TTG TTG TC	173 bp	0.2 µM		30
	R	CGC GTT GAG AAG CTG AGG TG				
Multiplex PCR 3						
aphA1	F	ATG GGC TCG CGA TAA TGT C	634 bp	0.4 µM	55°C	30
	R	CTC ACC GAG GCA GTT CCA T				
aphA2	F	GAT TGA ACA AGA TGG ATT GC	347 bp	0.1 µM		30
	R	CCA TGA TGG ATA CTT TCT CG				
Multiplex PCR 4						
sul1	F	CGG CGT GGG CTA CCT GAA CG	433 bp	0.2 µM	66°C	30
	R	GCC GAT CGC GTG AAG TTC CG				
sul2	F	CGG CAT CGT CAA CAT AAC CT	721 bp	0.3 µM		30
	R	TGT GCG GAT GAA GTC AGC TC				
Multiplex PCR 5						
bla $_{TEM}$	F	TTAACTGGCGAACTACTTAC	247 bp	0.2 µM	55°C	30
	R	GTCTATTTCGTTCATCCATA				
bla $_{CMY-2}$	F	GACAGCCTCTTTCTCCACA	1,000 bp	0.2 µM		30
	R	TGGACACGAAGGCTACGTA				
bla $_{CTX-M}$	F	TTTGCGATGTGCAGTACCAGTAA	544 bp	0.2 µM		31
	R	CGATATCGTTGGTGGTGCCATA				
PCR 1						
dfrA14	F	ATR GCT GCG AAA GCG AAA AA	455 bp	0.5 µM	47°C	7
	R	CCC TTT TTC CAA ATT TGA TAG C				
PCR 2						
intI1	F	GCC TTG CTG TTC TTC TAC GG	558 bp	0.25 µM	55°C	32
	R	GAT GCC TGC TTG TTC TAC GG				
PCR 3						
Variable region of class 1 integron						
	5' -CS	GGC ATC CAA GCA GCA AG	variable [(a)]	0.25 µM	55°C	32
	3' -CS	AAG CAG ACT TGA CCT GA				
PCR 4						
irp2	F	AAGGATTCGCTGTTACCGGAC	280 bp	0.5 µM	60°C	8
	R	TCGTCGGGCAGCGTTTCTTCT				
PCR 5						
pilV	F	ACAGGGGCAATACTTTCGTG	171 bp	0.5 µM	55°C	This study
	R	TGCTGGGCTCTCAACTACCT				
PCR 6						
traU	F	GTCGCAAGCAGTTTTGTTGA	167 bp	0.5 µM	55°C	This study
	R	TCATCCATTTCCCTGAAAGC				
PCR 7						
traW	F	CTGACAGCACGCCTAATGAA	241 bp	0.5 µM	55°C	This study
	R	TGCGCAGTGATTCTTACCAG				

(a) variable size depending on the inserted gene(s).

*F, Forward; R, Reverse
